# The AMIRO Social Robotics Framework: Deployment and Evaluation on the Pepper Robot

**DOI:** 10.3390/s20247271

**Published:** 2020-12-18

**Authors:** Alexandra Ștefania Ghiță, Alexandru Florin Gavril, Mihai Nan, Bilal Hoteit, Imad Alex Awada, Alexandru Sorici, Irina Georgiana Mocanu, Adina Magda Florea

**Affiliations:** Faculty of Automatic Control and Computers, University POLITEHNICA of Bucharest, 060042 Bucharest, Romania; stefania.a.ghita@upb.ro (A.Ș.G.); alexandru.gavril@cti.pub.ro (A.F.G.); mihai.nan@upb.ro (M.N.); bilal.hoteit@upb.ro (B.H.); alex.awada@upb.ro (I.A.A.); alexandru.sorici@upb.ro (A.S.); irina.mocanu@upb.ro (I.G.M.)

**Keywords:** social robots, robotic sensing, activity recognition, natural language processing, voice commands, active and assisted living

## Abstract

Recent studies in social robotics show that it can provide economic efficiency and growth in domains such as retail, entertainment, and active and assisted living (AAL). Recent work also highlights that users have the expectation of affordable social robotics platforms, providing focused and specific assistance in a robust manner. In this paper, we present the AMIRO social robotics framework, designed in a modular and robust way for assistive care scenarios. The framework includes robotic services for navigation, person detection and recognition, multi-lingual natural language interaction and dialogue management, as well as activity recognition and general behavior composition. We present AMIRO platform independent implementation based on a Robot Operating System (ROS). We focus on quantitative evaluations of each functionality module, providing discussions on their performance in different settings and the possible improvements. We showcase the deployment of the AMIRO framework on a popular social robotics platform—the Pepper robot—and present the experience of developing a complex user interaction scenario, employing all available functionality modules within AMIRO.

## 1. Introduction

Socially Assistive Robotics [1] refers to robots that are meant to assist people in a manner that focuses on social interactions (e.g., speaking, guiding, reminding, observing, and entertaining). Though physical interaction (e.g., carrying of objects) may be enabled by certain kinds of robot, it is not mandated by the mentioned definition.

One of the most focused domains of application for socially assistive robots (also referred to as *companion* robots) is that of supporting the elderly population, particularly people who are living alone or in care institutions, as well as those who are affected by medical conditions which warrant a closer monitoring of daily habits. The Active and Assisted Living (AAL) domain, which concerns itself with developing technology to support the needs of the aforementioned aging population, is therefore actively sustaining development of the capabilities of companion robots. In the AAL domain, *companion* robots are typically used to facilitate communication with the user and integration into a larger smart environment [2]. The robot may be tasked with facilitating telepresence, proactive notifications and reminders for medication or health related measurements, cognitive (e.g., through cognitive exergames) or informational support (e.g., information about weather forecasts, traffic or event updates), and interaction with a smart environment (e.g., vocal commands for turning on the lights, checking the status of smart appliances).

In their analysis of the impact of robotics on AAL [2], Payr et al. identify that primary user focus groups (i.e., the elderly users themselves) will usually regard companion robots as having a positive impact on people living alone and their relatives. The main benefit seems to be associated with the notion of safety and, interestingly, would more strongly improve the experience of elderly users which are not familiar with the regular usage of alternative technologies (e.g., PCs, tablets, and smartphones). Moreover, at the time of the Payr report, secondary caregivers (e.g., family and friends as well as professional care personnel) estimated lower adoption rates of the existing companion robot technology. However, they predicted continued increase in uptake of socially assistive robots, specifically as they provide the benefit of increased *independence* of seniors living alone at home. A motivation for this was highlighted in the market scenario envisioned in [2], where they show how acquisition costs of social robots can be offset when considering the time savings of professional caregivers, who are in high demand for performing simple monitoring tasks for many elderly individuals.

These analyses suggest, however, that a more proactive behavior (e.g., anticipation of needs and taking initiative in notifications) is expected on the part of socially assistive robots. Systems that aim to enable a useful companion robot are thus tasked with developing solutions that integrate functional building blocks from a wide range of perspectives, such as efficient navigation to the user whereabouts, improved perception abilities (e.g., object detection, person detection, user action recognition, and emotion recognition), improved voice-based interactions or improved integration with potentially pre-existing smart home solutions.

At the same time, research into user preferences regarding smart homes and service robots [3,4] suggests that elderly users prefer *on-demand* assistive functionality (i.e., decision-making is not autonomous, but within the control of the user). Users *still* favor robotic services that help them with physical tasks (e.g., cleaning, answering the door, and bringing objects), compared to primarily social ones. From the latter category, users are most interested in services that provide information, a sense of connection or increase their sense of safety (e.g., reminding of appointments or medication intake, personal health and home environment status check, and telepresence).

Such studies suggest that the interaction performance of social robots needs to improve. The focus on the physical service tasks is also an indication that users *expect* functionality aspects such as navigation, object detection, object handling, and natural language interactions to be *available* and *reliable* by default. Furthermore, while users may appreciate the novelty and usefulness of physical and social interaction capabilities of a robot, a lack of robustness in functionalities such as speech recognition, gesture recognition, navigation, and object handling will undermine the perception of usability and trustworthiness which are required by the user [5].

Recent research into social robotic platforms tends to keep count of the previously mentioned user preferences and develop systems exhibiting specific behaviors, which are evaluated in real user deployments. However, to the best of our knowledge, relatively few works provide quantitative, performance metric-based evaluations of all the individual functionalities of their social robotics system, using data streams obtained directly from the robot. It is therefore difficult for other research teams to obtain a baseline *expectation* of performance results, when deploying the system over a robotic platform and in more diverse environment setups.

Another relevant factor of analysis is the robot hardware itself, as well as its price. While some projects [6,7] develop custom built robots, in the attempt to keep the price tag below 15 k EUR, others (see, e.g., in [8]) focus on exploiting the advantages of more powerful, but far more expensive robots, selling for upward of 35k EUR.

Considering the above-mentioned aspects, in our own research we decided to develop a social robotics solution based on a platform that was designed specifically for social interactions, which has sold 12 k units in Europe as of May 2018 [9] and whose price stays within the 14–20 k EUR margin, depending on the financing scheme: the Pepper robot (https://www.softbankrobotics.com/emea/en/pepper/). Furthermore, we focus on addressing three research aspects which are of relevance in the social robotics domain. Our contributions in this paper refer to the following.

1. Developing a social robotics platform, called *AMIRO* (for AMbIent RObotics), deployable on the Pepper robot, but *necessarily* generalizable to any Robot Operating System (ROS) (https://www.ros.org/) compatible robot. The developed platform operates on an architecture that follows the recent trends of edge- and cloud-based robotics. It enables a comprehensive set of functionality modules that facilitate complex behavior composition: (i) navigation and obstacle avoidance; (ii) person recognition and coordinate estimation; (iii) human activity recognition; (iv) speech recognition, command processing, and dialogue management; (v) integration with smart environments; and (vi) belief-desire-intent similar composition and management of robot behaviors. Our effort focuses on developing a ROS-based social robotics framework performing the *integration* of existing and validated state-of-the-art models and platforms. We develop interfacing capabilities which allow for composition of individual module functionality into complex behaviors.
2. While many works in the literature focus on evaluating social robotics systems as a whole, few of them provide a *reference point* for the performance of each individual capability, using data streams collected directly from the robot platform. In particular, these observations stand true all the more in the case of the Pepper robot. One important contribution is therefore that of performing quantifiable tests of each individual functionality module, based on datasets collected directly from the Pepper robot in diverse laboratory setups. The documented experiments provide a reference for the robustness of the developed social robotics platform and inform on the advantages and downsides inherent to the Pepper hardware or perfectible in our current implementation.
3. Qualitative evaluation of the developed framework by means of a scenario highlighting the interplay of all available functionality modules. The scenario involves receiving of a notification from an external health management service, proactive search and navigation towards the user, natural language interaction, and activity recognition (drinking water).

We organize the presentation of these contributions in the following way. In Section 2, we position AMIRO with respect to related work by analyzing and comparing existing social robotics frameworks, as well as state-of-the-art developments for each the available functionality modules in AMIRO. Section 3 describes the AMIRO platform in terms of overall architecture and details of each provided functionality module. The performance analysis of the AMIRO functionality modules is presented in Section 4, while the composition of these modules into a complex scenario is evaluated in Section 5. We conclude the paper and specify directions of future work in Section 6.

## 2. Related Work

Given the motivation outlined in the introduction, the social robotics domain has seen an increased support in recent years. Social robots are designed to interact with people in a natural way, having intention like humans.

### 2.1. Social Robotics Systems

A number of research projects have developed solutions, comprising a diverse set of functionalities, from more specific ones [10,11], to general systems [7,8,12], to initiatives (e.g., STRANDS [13]) that support development of technologies for long-term autonomy of robots.

Our focus is on providing a framework for general social robotics (i.e., comprehensive set of capabilities). Our review of related work aims to analyze aspects related to the openness and extensibility of the supporting core platforms, the set of core functionalities enabled, performance metric-based testing of each enabled functionality, as well as system wide testing of interaction scenarios. Our findings are summarized in Table 1. We proceed in what follows with a more detailed description of reviewed frameworks and systems.

NAOqi and the visual IDE Choregraphe [14], which is based on it, are the default frameworks for composing social interaction capabilities on the Pepper robot, which we use in our deployment. NAOqi provides rich development capabilities, but its downside is that its application is limited to Softbank’s robots. The developer is also tied in exploiting the builtin modules such as user detection, speech recognition or navigation. On the other hand, the AMIRO framework places its foundation in ROS, allowing its deployment over a much larger base of robots, as well as the interfacing with state-of-the-art modules for key functionalities (cf. Section 3). Other frameworks are more focused on the social interactions. An example is Interaction Composer [15], offering similar behavior-based programming to AMIRO, while being suitable for non-technical users. It can, in theory, be used on top of ROS, yet this is not intrinsically supported. The RADIO project [11] comes close to the technical integration capabilities that we envision in our own research. While the framework enables ROS-based interfaces that connect a robotic unit with ambient monitoring sensors, its main application area is that of multimodal (e.g., vision and sound) detection and monitoring of Activities of Daily Living (ADLs). Furthermore, much of the project is focused on the technical integration itself. In contrast, our research is focused also on providing a means for goal-driven development of the robot life cycle and enabling a fuller range of possible interactions.

Apart from frameworks that enable development of social robotics solutions, there are a number of developed *systems* whose objectives and functionality items are similar to the ones enabled by AMIRO. We continue the related work overview by examining such systems in terms of their provided functionality set, existing performance tests, as well as deployment scenarios and their results. We then provide a closer look at recent state-of-the-art for each of the functionality modules implemented in AMIRO.

The Robotic Activity Support (RAS) [6] develops a system that links smart home technologies with robots to provide assistance in carrying out daily activities. Authors test specific functionality modules (e.g., object detection and personal activity monitoring) of their social robot platform in a lab setting involving 26 younger adults. The object detection evaluation is based on a comparison between popular pretrained neural network architectures such as R-CNN, R-FCN, or SSD, and obtains good precision scores for objects one might typically find in a household (e.g., dog leash, keys, cup, umbrella, and plant). However, the tested networks perform poorly in detecting people, with a precision of less than 50%. Currently, the robot has a single principal behavior, that of observing users while they carry out an activity and noticing if they execute the steps of the activity in the correct order (e.g., detect missing steps or order switching). Questionnaires collected from participants rate the robot user interface (which uses a tablet) as favorable, but the authors report that users had a neutral rating of the overall usability of the activity monitoring and support system offered by the robot. One point of needed improvement is the response time of the robot (e.g., navigating to the user and displaying a video of the required sequence of steps to perform the activity) when an error in performing an activity has been detected. In contrast to our solution, the RAS system is more specific and, as currently described, does not facilitate arbitrary behavior composition, natural language interaction or longer distance navigation.

The EnrichMe project [8] targeted aiding the independent living of single older adults via smart-home, robotics, and web technologies. The project used a custom version of the TIAGo Iron robot which had three main behaviors: locating lost objects in a home, activity monitoring, and abnormal situation detection. Object localization is performed by placing cheap RFID tags on specific objects of interest (e.g., remote control, keyring, and glasses) and confidence region-based algorithm (constructed using tag grid maps) to assign the most probable object to the current robot position. Human detection and tracking is based on a fused information model that combines data from a laser scanner that identifies legs of people, an image-based upper body detection method and whole body thermal imaging. The activity recognition is limited to detecting basic actions (e.g., walking, standing still, and moving hands) based on analysis of 5 s intervals, while the anomaly detection can only specify if a person spends an abnormal time in a given room, as compared to prior statistics [16]. The system was tested qualitatively in six deployment scenarios across the UK, Greece, and Poland by a total of 11 users, with satisfying results, specifically with respect to user interactions with the robot tablet applications (e.g., cognitive games and weather service). Object detection had more faults in the live deployments, due in most part to the predefined path that the robot could use in each home, which was too far from the objects to provide enough confidence on the assignment of an object to the current robot region based on the RF signal. The project also performed quantitative evaluations of each functionality module in a laboratory setting. A general performance metric for the RFID-based object localization was missing. Person identification has a precision of over 90% (although F1 score is low—25%), while person re-identification achieves a less than 50% precision rate and a ~40% recall rate. Failures appear in cases of people with similar body shapes or when they are affected by partial occlusions. The robot lifecycle is governed by a Hybrid Behavior Based Architecture (HBBA), which is similar in design to the Belief-Desire-Intent (BDI) model, and is implemented as a collection of Robot Operating System (ROS) nodes. The EnrichMe project is overall most similar in design principles, functionality modules, testing procedures and qualitative evaluations to our own solution for social robotics. However, it is missing a more comprehensive test of navigation capabilities, as well as means to detect more diverse user actions (e.g., working on a smartphone, sitting and typing on a keyboard, and drinking from a cup).

The SocialRobot project [7] focused on developing a custom robot for elderly care, focusing on essential aspects of care provisioning and on affordability. The system is based on a service-oriented architecture and the specific services implemented include navigation, people detection and face recognition, audio-based emotion recognition, simple word detection, as well as a tablet-based UI that can launch touch-based applications or Skype calls. The robot has been tested qualitatively in a care center in the Netherlands using a simple behavior whereby the robot wanders in a defined perimeter and asks users how it can be of assistance (e.g., take picture, suggest activity, show agenda, and Skype call) when approached by a person. A questionnaire filled in by 30 users of the senior center shows a good evaluation of the robot in terms of ease of use, usefulness, support for independence, and activity or feeling of companionship). However, the authors do not report a more systematic performance of individual functionality modules (neither in the live deployment, nor in a lab setting).

In our own research we decide to use and test the Pepper social robot (https://www.softbankrobotics.com/emea/en/pepper/), specifically the 1.6 version, to which we made a simple hardware modification. Pepper is gaining more and more traction as a friendly social interaction robot, having sold 12 k units in Europe as of May 2018. Selling at 14–15 k USD, it comes close to the price tag of solutions used in the RAS and SocialRobot projects. While the robot ships with a number of functionalities (e.g., face detection, people tracking, dialogue, and tablet-based interaction) which allows for defining simple interaction scenarios, we expand the capabilities of the robot by developing functionality modules for: improved navigation, improved user identification and tracking, improved speech recognition and custom command understanding, human activity recognition, as well as services for integrating with a smart environment. All of these services are quantitatively tested in a lab setting, under data streams coming directly from the robot.

### 2.2. Navigation and Obstacle Avoidance

Indoor robot navigation is inspired, like many other functionality domains, by the way humans are performing it [17]. Most robotic navigation systems use Simultaneous Localization and Mapping (SLAM) to solve the problem of building a map of the environment while moving, and localizing the robot inside of the created map. Trajectory planning systems provide the robot with the ability of moving to a valid target on the map. SLAM can be implemented in various ways, given the specifics of hardware and sensing used for distance or similarity measurement. The quality of the sensors highly influences the quality of the environment representation.

With respect to the Pepper robot, which is used for our AMIRO platform deployment and testing, the authors of [18] report on usage of Pepper’s own range sensors (LiDAR installed in the robot mobile base) to perform both mapping, localization, and planning using frameworks available in ROS (e.g., *slam_gmapping*, *cartographer*, and *DWA* global and local planners). Results suggest usable maps obtained by *gmapper* to an extensive cost of manual tuning of parameters. However, localization in a SLAM created map suffers. This leads to good navigation in straight lines, but to problems when taking corners. A suggestion to use more than the ranger sensors is made as future work objective in [18]. This suggestion is followed by the work in [19] which uses the RGB and depth sensors of Pepper to perform visual SLAM (specifically, an adapted version of the ORB-SLAM algorithm [20]), using the range sensors only for obstacle avoidance during navigation. However, in this case SLAM takes longer to complete and the approach has difficulty in estimating a correct localization in visually feature-poor environments (e.g., a large hall), given the narrow field of view of the RGB camera installed on Pepper. In an attempt to make navigation more interactive, the authors of [21] develop an human–robot interaction (HRI)-centered approach, whereby the local planner of the *DWA* ROS planning framework is extended to include a social interaction in case the robot path is blocked by a human. The social interaction consists in the asking the human to move out of the way, if the local planner cannot find a quick way around the person. The authors implement mapping and localization using the standard ROS (*gmapping*, *amcl*) and Pepper’s range sensors, but they report only on initial deployment tests in a narrow environment (where the lasers onboard Pepper can still provide accurate results).

Recognizing the above-mentioned limitations of the onboard sensors of Pepper, in our own previous work we develop enhanced localization and navigation capabilities by installing an additional LiDAR on the robot and fine tuning the *amcl* parameters [22]. This enables navigation through doors, wide corridors, and desk-dense lab environments. We provide some additional details on the implementation of the navigation component in Section 3.4.

### 2.3. Person Recognition and Coordinate Estimation

In Wang et al. [23] two mathematical methods are proposed to solve the coordinate estimation problem: linear observer and nonlinear observer models. The proposed linear observed model uses a geometrical approach to compute the coordinates estimation based on the linear and angular velocities of the camera. However, because the velocities are only approximations of the real values, the estimations are not accurate. The nonlinear observer model is introduced for more accurate estimations, but considering the complexity of the mathematics behind it, two simplified situations were analyzed: the linear velocity of the robot is known and one component of the interest point is known. In both simplified situations, the nonlinear observer can be designed to obtain a coordinate estimation.

Chao et al. [24] proposed a mathematical method to compute the 3D position of a target recorded by the camera of a mobile robot. The method uses the calibrated parameters of the camera, the odometry of the robot, alongside the triangulation principle to estimate a coordinate. The robot moves and acquires multiple images from different angles for the same target in order to obtain an accurate estimation. The evaluation was done by moving a tablet instead of a robotic platform, which does not take into consideration the reliability of the odometry of the robot. The main problem of this method is that it can only be applied on static objects, as the image acquirement process takes a certain amount of time. As people move in the environment, the acquisition of images can be compromised and the estimation of the 3D coordinates may give incorrect results.

In contrast to these works, our solution uses robot pose information, person bounding box information, and depth sensor input to reliably approximate the *orientation* of the user with respect to the robot, and to provide best effort estimates of the *distance* toward the user. This method proves simple and efficient when the movement *towards* a user is done in steps, based on distance intervals. We explore the details in Section 3.3.

### 2.4. Human Activity Recognition (HAR)

The problem of recognizing human actions has great practical applicability which made it become one of the most attractive research fields. Many factors can define an action, such as the posture of the person performing it, the objects with which the person interacts, the environment in which the action is performed, the speed with which it is performed, but also factors related to the quality of the data collected. All this makes the problem of recognizing human actions a complex and challenging one, but even so, recently proposed deep learning-based solutions have obtained very good results on the known benchmarks. Some of them [25,26,27] have been integrated into robotic systems in order to improve the interaction between people and robot, turning the robot into a proactive one, capable of understanding what action a person performs.

Rezazadegan et al. explain how techniques proposed for HAR should be adapted for use on a robot [28]. They start from the fact that most of the datasets used for HAR are recorded using static cameras, while the robot is a mobile platform that can move during the action. To solve this challenge, they proposed a method that works in two steps. In a first stage, generic action region proposals are generated, which have a good potential to locate one human action in unconstrained videos, regardless of camera motion. In a second step, they use convolutional neural networks to extract features to classify the action. They also proposed two datasets: one achieved through a careful composition of camera footage and the other through acquisition by a mobile robot.

Chiang et al. [29] proposed a culture-aware HAR system composed of two important components: an ontology which aims to link activities with culture-specific properties extracted from the context by experts and a module based on Bayesian Networks to expand the ontology with probabilistic reasoning and link the knowledge therein with the recognition results provided by the HAR system. To test this proposed system, they used two types of scenarios: one offline with images collected from the Internet and one online with images collected using the Pepper robot. An important aspect, highlighted by them based on the experimental results obtained, is related to the importance of selecting the appropriate distances and orientations for the robot to capture all the user’s gestures.

Recently, various types of methods have been proposed that have obtained good results for this problem. The best-performing solutions for methods based on 3D skeleton joints [30,31,32,33] combining the advantages of two important types of networks: Temporal Convolutional Neural Network [34] used to capture temporal dependencies and Graph Convolutional Neural Network [35] used to model spatial dependencies. In contrast, the solution we propose, starting from the analyzed models and the observations that we presented in the previous works [36,37], is based on a multi-stage architecture based on linear layers used to extract features and long short-term memory layers used to model the sequence of frames to obtain the correct action. Thus, our contribution consists in proposing a model with a relatively small number of parameters, which can be used in real-time scenarios, but with the ability to differentiate between similar actions. This last aspect is possible due to the improvement of the prediction from one stage to another.

### 2.5. Speech Recognition, Command Processing, and Dialogue Management

Verbal interaction is the most successful form of communication between humans. To make the interactions more natural, attractive, and easy for the users, any social robot should be able to interact with the user thorough verbal interactions. During the last two decades, major progress has been achieved in the different aspects of the human–machine verbal interactions, such as in the Automatic Speech Recognition (ASR), Natural Languages Understanding (NLU), Dialogue Management (DM), and speech syntheses (TTS) fields. This progress enables developers to create complex and reliable speech interaction systems.

Many researchers addressed the different fields of the human–machine verbal interactions, and they offered different solutions for the issues that were identified such as in [38,39,40] for the ASR, in [41] for the NLU, in [42] for the DM, and in [43] for the TTS.

Potbora el al. [44] present a detailed concept of an architecture for human–robot interaction systems that includes speech interactions with the Pepper robot. The authors found that the build-in speech recognition module of Pepper (which is based on the NUANCE engine) is limited as it supports only few languages and the embedded recognition-library can recognize only phrases that are include in a predefined set. Therefore, the authors aimed to develop their own speech recognition module in order to fulfill the requirements of a real conversation. Perera et al. [45] found some limitations with the built-in speech recognition module of Pepper, and they used Pepper’s tablet to bypass those limitations.

Recognizing these limitations ourselves, our solution consists in a cloud-based pipeline of services that provide ASR, NLU, DM, and TTS functionality for a consistent (and extendable) set of languages. One added benefit of our solution and its integration interface is that it is not specific to a robotic platform deployment and can be used separately in other applications requiring voice-based interactions.

### 2.6. Integration with Smart Environments

The smart environments are nowadays more and more evolved with various ways of integrating the sensors and actuators in a centralized or decentralized way. Commercial solutions are available, yet most of them are proprietary and offer little support in customizing them.

Most of the existing systems for smart environment integration are focusing on integrating the robot with standardized platforms for smart environments. This is usually performed using centralized gateways which integrate the sensors and offer remote access to the data. Depending on the use case, the robot can be integrated as a source of information [46,47] or be used as an interface to interact with and gather data from the smart system [48].

In [49], the Pepper Robot is integrated with a fully functional smart home called iHouse. The system uses the NaoQi framework to perform speech to text, then Api.ai as a natural language processing tool to classify the utterance and match it to an action. The uAAL (universAAL) platform is then used to facilitate the communication between the robot and the smart home through the uAAL middleware. All the smart devices are integrated using the ECHONET protocol. The ECHONET objects are translated into uAAL resources (ontologies, objects) and passed back to robot through the middleware. The proposed solution is very robust and make use of standardised open source platforms and systems.

Similar to the work in [47], our smart environment integration solution considers the robotic platform as a means to interact with and collect data from the environment. We make it easy to use the smart environment integration in robotics projects by providing a mechanism to transform external data sources in ROS nodes; thus, keeping a uniform (topic based) data communication and actuation process within the AMIRO framework.

### 2.7. Behavior Management

Behavior management is a central component of any social robotics platform as it builds the robot life cycle, controlling the way in which the robot behaves in different environment and user interaction situations. Its importance stems from the fact the active and fail-safe behaviors it composes contribute directly to the sense of naturalness perceived by human users. Nocentini et al. [50] provide a comprehensive survey of behavioral models for social robots, covering topics of cognitive architectures, behavioral adaptation, and empathy in very diverse (but also very specific) use cases and deployments. The authors highlight the fact that establishing an HRI benchmark that comes close to usual human–human interaction is an enormous challenge, which is why the papers in their survey focus on very specific use cases, wherein both classical planning, as well as learning frameworks, are used to define robot behavior.

The specific use case deployments are in keeping with observations made by user preference assessment research [3,5] which notice that users prefer social robotics solutions that have predictable and robust (even if reduced) behaviors, that fulfill a concrete user need, over general interaction and proactive robot behavior which does not work reliably. This is in keeping with the uncanny valley effect [51], which is noticeable in recent general solutions for social robotics.

The solutions discussed at the beginning of this section [6,8] implement behavior management using state machine [6] or Belief Desire Intent (BDI)-like frameworks [8], that help them implement specific user assistance scenarios (e.g., detecting erroneously executed activities [6]).

In a manner similar to the mentioned works, our own behavior management frameworks [22] works by constructing a basic behavior execution graph, whereby nodes are linked via success and failure edges. Execution of specific basic behaviors can be preempted when more important *desires* arise. The importance and order of basic behavior execution are controlled via a priority queue mechanism. This allows us to implement the sequence of actions needed for the assistance scenario presented in Section 5. We give more details of its implementation, as well as future extension possibilities in Section 3.7.

## 3. Proposed Framework

The proposed framework addresses the major requirements for a social assistive robot, while maintaining a high degree of modularity in building and integrating new modules inside the system. The main modules of the system are listed below.

- Navigation and obstacle avoidance
- Person recognition and coordinate estimation
- Human activity recognition
- Speech recognition and dialogue management
- Integration with smart environments
- Behavior composition

The *dialogue*, *person recognition*, *robot localization*, and *smart environment integration* modules are always active and can be run asynchronously, as the robot can perceive voice commands, humans, and localize itself while performing any other tasks. The *navigation and obstacle avoidance* and the *activity recognition* modules require activation by the other modules and can be run one at the time. The behavior composition (planning) module is responsible with activating and deactivating the other modules based on the current objective.

The system can be triggered to act by commands sent either by the dialogue module or by any objective currently active in the behavior composition module. The dialogue module can start certain scenarios such as finding a person, showing the health status or interacting with the smart environment.

### 3.1. Architecture and Implementation

The architecture of the system is built on top of the ROS framework. Each module offers a set of ROS publishers and subscribers which are integrated in the system architecture. From a module deployment perspective, the AMIRO architecture distributes its services on machines running in the cloud or constituting the cloud-edge (https://github.com/aimas-upb/amiro/, https://github.com/aimas-upb/sparc).

**Figure 1:**
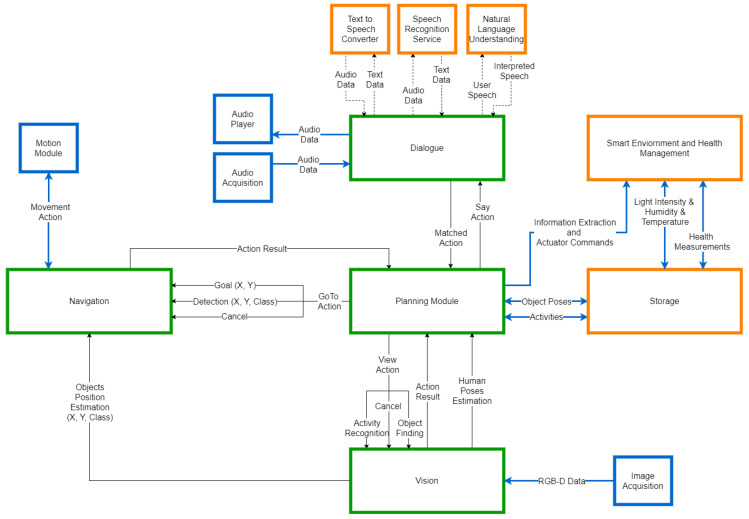
AMIRO System Architecture—block diagram. The green boxes represent the cloud-edge components, orange boxes represent cloud services, and blue boxes represent modules operating on the robot. If the arrow between boxes is black, then it represents an internal call, if is dotted it represents a call to a cloud service and if it is blue arrows then it symbolizes a communication through ROS.

The building block services of the AMIRO platform and the principle information exchange between them are shown in Figure 1. The main components of the system and the ones which constitute the cloud-edge are represented with green border. The orange squares symbolize the services that are running on cloud, while the blue ones are the modules operating on the robot. The information exchanged between the cloud-edge components and the rest of the system is transmitted through ROS channels, while the information between cloud-edge components is directly passed as function parameters inside the main program.

The advantage of this architecture and its deployment is the fact that each node can be run on separate machines, allowing the separation of concerns and facilitating the deployment of the system. As such, the robotic platform is used for data acquisition and actuation through the ROS topics. The ROS Master runs on a cloud edge device (e.g., a local server), together with the ROS nodes responsible for navigation, vision, and behavior composition (planning) modules, given that these services require a higher data traffic and more computing resources to provide effective results. The Storage, Smart Environment, and Health Management, as well as the services in the dialogue module are running in the cloud, and their implementation and interfacing options allow them to run as separate services for other applications, other than the AMIRO platform.

The deployment of the system is justified by the real-time requirements of the project. The fast response of the system is conditioned by the intrinsic capabilities of the robot and the volume of the data to be processed. The techniques used in the vision component require strong computational capabilities, while the robot used to test the framework did not match those requirements. Moreover, the framework should be easy to integrate with multiple robots, so it should not impose computational requirements on the robotic platform. These are the main reasons behind the choice of the cloud-edge modules. We chose to use local data processing instead of cloud processing by taking into consideration the amount of information needed to be transmitted over the internet. To have fast responses, the bandwidth of the internet connection should be large enough to not add latency while passing visual information. This is also the case for the navigation component, as the experiments proved that the bandwidth was a limitation for higher distances. For the other modules, we decided to utilize cloud connection, as there are few requirements in terms of internet connection, given the small amount of exchanged data.

The Behavior Composition (Planning) module is at the center of the system, subscribed to the central Storage for data acquisition and publishing commands to all the other modules. The planning module runs directly on the machine hosting the ROS master node.

The Navigation module is responsible with robot movements inside the environment and is triggered by the Planning module. The module performs data acquisition and the processing of the SLAM algorithms on a cloud edge node (e.g., a local server).

The Vision module integrates the object recognition, object segmentation, and the activity recognition components. When an object detection occurs, the 2D position estimates are forwarded to the Navigation module, which computes the 3D position on the map, based on the current robot estimated pose. As most of the used algorithms require powerful parallel computation and real-time data acquisition from the cameras, the module runs directly on the machine hosting the ROS master node.

The Dialogue module can send new tasks to the Planning module based on the current dialogue with the user and offer Text-to-Speech capabilities when required. The module can use any microphone input and is deployed as a separate service, running in the cloud. This allows it to be easily used for other speech-based applications.

The Smart Environment and Health Management module gathers user health (blood pressure, heart rate, steps, and sleep), environment information (room temperature, humidity, and luminosity), and provides the necessary topics to actuate different smart devices (e.g., smart lighting and smart blinds). The information can be requested by the Planning module to be uttered or displayed to the user. The module can also insert new tasks to the Planning module based on the reminders or calendar events of the user. This module is also deployed as a cloud-based service allowing it to be easily integrated in other systems. The module also provides a web interface facilitating classical interactions with the AMIRO platform.

### 3.2. Speech Recognition, Command Processing, and Dialogue Management

The ability to interact in natural language is essential in developing a social robotic platform. It makes the interaction between the user and the robot easier, more natural and more attractive.

The module is composed of five components: Audio Preprocessing, Automatic Speech Recognition (ASR), Natural Language Understanding (NLU), Dialogue Management (DM), and Text-to-Speech (TTS) Synthesis. It supports three languages: English, Romanian, and French.

Audio Preprocessing: This component is responsible with extracting the data that is related to the dialogue module from the profile of each user as well as from the robot’s settings such as the speech language, the voice that the user prefers to be used in the TTS component for the generated audio files, the bit depth of the audio as well as other useful data that ensure the best results for the dialogue component.

Automatic Speech Recognition (ASR): This component is responsible to convert the speech command received from the user to text. The component uses the Google Cloud Speech-to-Text API (https://cloud.google.com/speech-to-text/) as the speech recognition engine.

**Figure 2:**
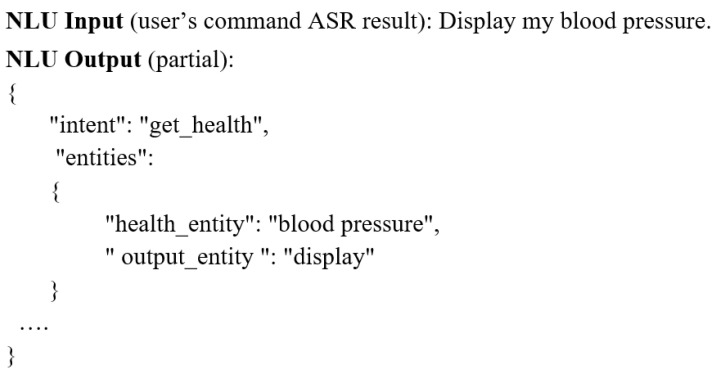
The input and output of the NLU.

Natural Language Understanding (NLU): This component is responsible for generating a machine-readable representation which allows the robot to understand the command of the user. The component receives a text from the ASR from which it extracts and classifies different intents and entities. Figure 2 illustrates the input and output of the component. In this example, the user asks from the system, through a speech command, to display his/her blood pressure. The user used the “Display my blood pressure” command, but he/she could have used other alternative commands such as “show my blood pressure”, “display my blood pressure measurements”, and “show my systolic and diastolic pressures”. The NLU analyses the transcribed text of the voice command (that is received from the ASR) and extracts the intent and entities of the user. In the illustrated example, the intent is “get_health” and the entities are the “health_entity” (blood pressure) and the “output_entity” (display). In the case in which the user gives the same command without specifying an “output_entity” such as in the case of the “What is my blood pressure”, the NLU will consider the default value of the entity (visual and phonetic or in other words display and say). In addition, in the NLU component, any mistakes that may happen during the ASR step are corrected. To make this possible, for each language, multiple intents were created to which different entities have been associated. The component uses the wit.ai (https://wit.ai/) API as the NLU solution.

**Figure 3:**
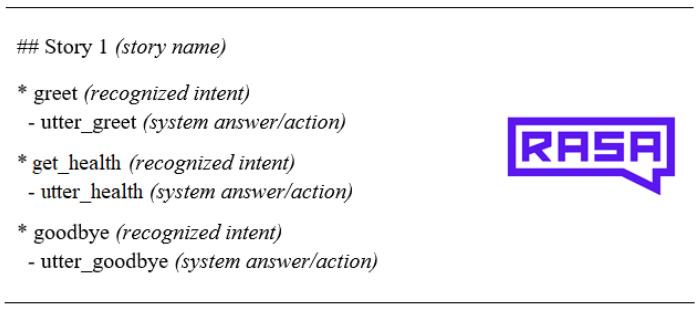
A RASA story.

Dialogue Management (DM): This component is responsible for maintaining the state and flow of the current conversation. This module decides what should be the answer of the robot according to the machine-readable representation received from the NLU. In addition, the component maintains a history of the current interaction, as the information that is required for an action to be executed by the robot may be provided by the user through a single interaction or through multiple interactions. To achieve this, RASA (https://rasa.com/) is used. In RASA, several stories are created, each story consisting of a series of interactions between the user and the robot as illustrated in Figure 3.

Text-to-Speech (TTS) Synthesis: This component is responsible to convert the text received from the DM into an artificial human speech audio file that will be heard over the phonetic output the robot. For the English and French languages the Google Cloud Text-to-Speech API (https://cloud.google.com/text-to-speech/) is used as the TTS engine, while for the Romanian language, the Responsive Voice API (https://responsivevoice.org/) is used as the TTS engine.

The output of the system is translated to a command sent to the planning module. The planning module matches the dialogue command by intent and entity to a certain action that the robot must take.

### 3.3. Person Recognition and Coordinate Estimation

The 3D coordinates estimation module is approximating the real position of a target relatively to the current position of the robot. It associates a 3D position for every person detected by the object detector, using the visual information acquired by the sensors.

The detection component of the module uses YOLOv3 system [52] to compute bounding boxes of the people in the images and FaceNet architecture [53] to recognize the faces. The information extracted by the two methods is aggregated in order to obtain a more accurate representation of the individuals in the images, for a more precise estimation of their 3D coordinates.

The computation of the estimated position integrates the position of the detection in the RGB image, the distance to the detected area observed by the depth sensor and the values of the angles of the RGB camera with respect to the vertical and horizontal axes. The approximated coordinates are calculated according to the following formulas.

$\alpha =hfov_{camera}*\left(\left(width_{image}/2-x_{image\_detection}\right)/\left(width_{image}/2\right)\right)$
$\beta =vfov_{camera}*\left(\left(y_{image\_detection}-height_{image}/2\right)/\left(height_{image}/2\right)\right)$
$x=d*cos(\alpha +yaw\_angle_{robot_{h}ead})$
$y=d*sin(\alpha +yaw\_angle_{robot_{h}ead})$
$z=d*sin(\beta +pitch\_angle_{robot_{h}ead})$

**Figure 4:**
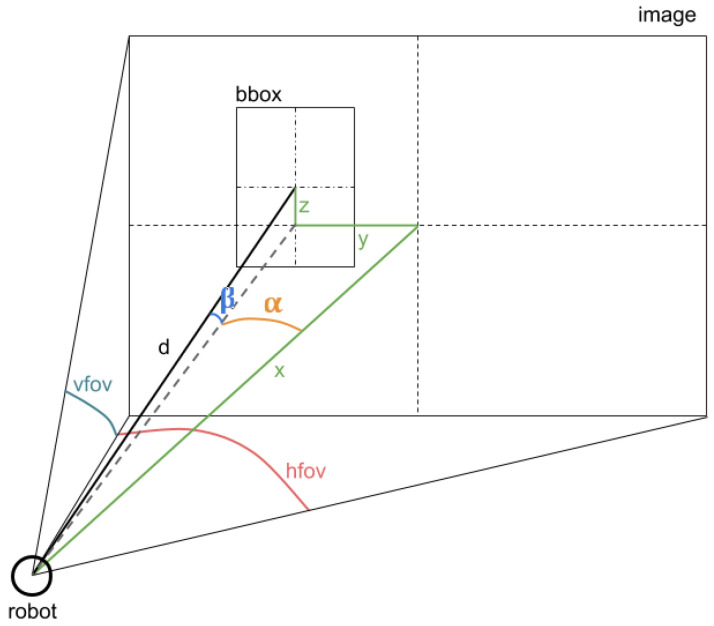
Graphical representation of variables in the formulas for the estimation of the 3D coordinates of detected people. (*x*, *y*, *z*) are the estimated person coordinates with the robot *map* frame. α and β are the angles of the detected person with respect to the position of the sensor. $vfov_{camera}$ and $hfov_{camera}$ are maximum angles for vertical and horizontal field of view, respectively. *d* is the distance to the detected person as perceived by the depth sensor.

In the above formulas, α and β are additional computed values which represent the angles of the detected person with respect to the position of the sensor. The constants $vfov_{camera}$ and $hfov_{camera}$ symbolizes the maximum angles of the field of view of the RGB sensor on the vertical and horizontal axes. For the computation of the 3D coordinates (*x*, *y*, *z*), *d* represents the distance to the detected person acquired by the depth sensor, while the yaw_angle and pitch_angle variables indicate the Euler angles of the sensors when recording the RGB-D images. A clearer representation of the variables used in the formulas is presented in Figure 4. A detailed section about the mathematics behind the method can be found in [54].

### 3.4. Navigation and Obstacle Avoidance Module

Robot navigation is required in any robotic assistive scenario. The ability to have the platform move to the person in need can provide a much better assistance. The robotic platform must be able to move to a person while being aware of any obstacles on the trajectory. Safety is a very important aspect when having a robot interacting in a live environment with other humans; therefore, the robot must take decisions fast and reliably when moving.

The main aspects of robotic navigation are being able to detect obstacles, planning the path accordingly and being able to localize oneself inside the environment. The detection of obstacles is usually performed by mapping the surroundings with distance measuring sensors. The localization is then performed on the mapped environment by matching the sensor input to a position on the map.

As the navigation module is required by all the other modules integrated in the system, it provides the necessary ROS topics to allow movement to a certain coordinate on the map, forward or rotational movements and emergency stop.

The navigation module is an external system composed of a 360°RP1 Lidar (https://www.slamtec.com/en/Lidar/A1/) attached to an acquisition board, running as an edge ROS node which exposes the laser scans on a ROS topic. The laser scans are then taken in by the proposed system and integrated into the SLAM module. The SLAM module was realized based on Hector SLAM [55] and tuned to work with the equipment. This was preferable to the usage of the default robot sensors due to the limitations in range. This has the benefit of having an external navigation system which can be easily removed and placed on a different robot with minimum configuration effort. More details about the general architecture of the system can be found in [22].

### 3.5. Activity Recognition

To have a robot that can interact with humans, the robot must identify the actions performed by the person it is monitoring or to whom it must send a notification. Thus, the module for recognizing human activities was included in the proposed framework for the Pepper robot, to obtain a socially assistive robot.

Given that the problem of human action recognition is a very popular one and it has great practical applicability, several datasets have been developed. One of these is NTU RGB+D [56,57] which includes samples for 120 actions. One of the modalities provided by this dataset is composed of skeletal data collected using Microsoft Kinect v2. In the module for recognizing human activities for the Pepper robot, we decided to use this format for the data that characterized a human action. As the robot is not equipped with such a sensor, we had to find a solution to collect the data in a similar format. Thus, we decided to use a pretrained OpenPose [58] model to determine the human posture, in 2D format, starting from the RGB images collected by the robot. To extend these coordinates to 3D ones, it was necessary to make a spatial alignment of the RGB images with the depth maps, as well as a temporal alignment, because the frame rates for the two cameras of the robot were different. In order to obtain a module capable of correctly recognizing human action regardless of the lengths of the segments that make up the subject’s posture, a normalization was applied. In order to achieve this normalization, some standard dimensions were calculated for each type of segment, averaging the dimensions in the samples included in the NTU RGB+D dataset [56,57].

**Figure 5:**
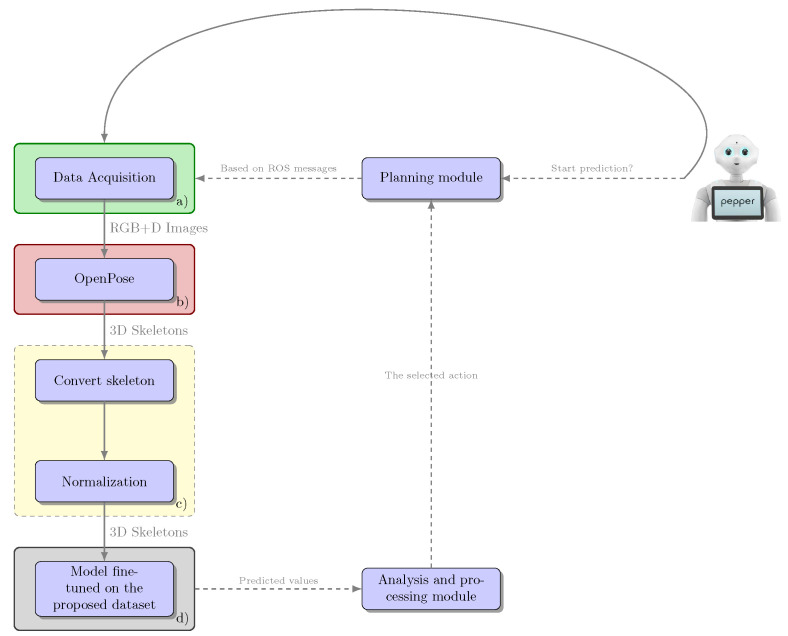
The architecture of the complete integration process of the human action recognition module.

**Figure 6:**
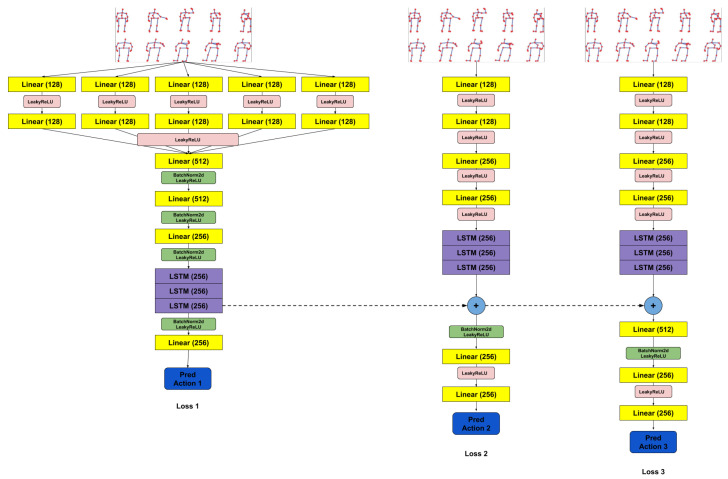
The architecture of the neural network used to recognize human action.

The pipeline of the entire integration process is shown in Figure 5, and the network architecture used as a human action classifier is presented in Figure 6. In the paper [36], we presented an architecture that failed to correctly differentiate similar actions (such as *drink water* and *sneeze/cough* or *hand waving* and *pointing to something*). Thus, we decided to propose a new extended architecture that contains two additional stages that receive as input the sequence processed with the coordinates of the skeletons but also takes into account information from the previous stage. Each stage uses a series of linear layers to extract features from the skeletal coordinate sequence and an LSTM network for analyzing these temporal sequences. A loss function was applied for each stage. The action classifier used was trained on the NTU RGB+D dataset [56] and then specialized on a dataset collected using the Pepper robot. The dataset collected with the Pepper robot contains a subset of eight actions considered to be relevant and challenging for a robot used as a personal assistant. Because there are very similar actions (e.g., *playing with phone/tablet* and *typing on a keyboard*), within this subset of selected actions, a complex model was needed to be able to differentiate the actions correctly. Thus, we proposed an architecture composed of three stages. After this classifier was trained, the result provided by stage three was used as the final prediction. The architecture of the classifier is an improved version of a model tested and analyzed in our previous work [37].

### 3.6. Smart Environment and Health Management

The smart environment interactions module is a plug-and-play module used to further increase the capabilities of the system and provide better care services. The capabilities to sense certain parameters inside the environment like humidity, temperature, and light intensity can help the system in performing automated tasks based on the human preferences. The interactions with the light switches, blinds, and other actuators inside the environment can further increase the comfort level of the human in need. Currently, the system integrates the actuating commands and can be triggered either by a voice command or by interacting with the user interface.

In contrast with most of the existing solutions, the module focuses on bringing the smart devices closer to the standardized frameworks for robotic interaction. The system facilitates the integration of additional sensors through custom-made ROS nodes and messages. Additional edge nodes can be integrated in the system by simply publishing the acquired data to the corresponding ROS topics using the predefined messages. This allows the system to gather data from any sensor placed inside the environment. In order to actuate additional edge nodes like smart lighting and smart blinds, the ROS nodes must have a unique name and offer the topic on which the system should send the corresponding message.

The health management module extracts available health information from the storage unit like heart rate, blood pressure measurements, steps, and sleep information. The module integrates various health sensors which are saved in the storage unit. The robot is then able to display the information to the end user. The system also integrates a notification mechanism which can trigger the robot to start an action. For example, when the user is required to take a blood pressure measurement, the robot can be triggered to navigate to the person position and remind him.

### 3.7. Behavior Composition

The guiding principle of the current behavior composition module is that of easiness and robustness in bringing together the basic behavior functionality aspects discussed in previous sections.

**Figure 7:**
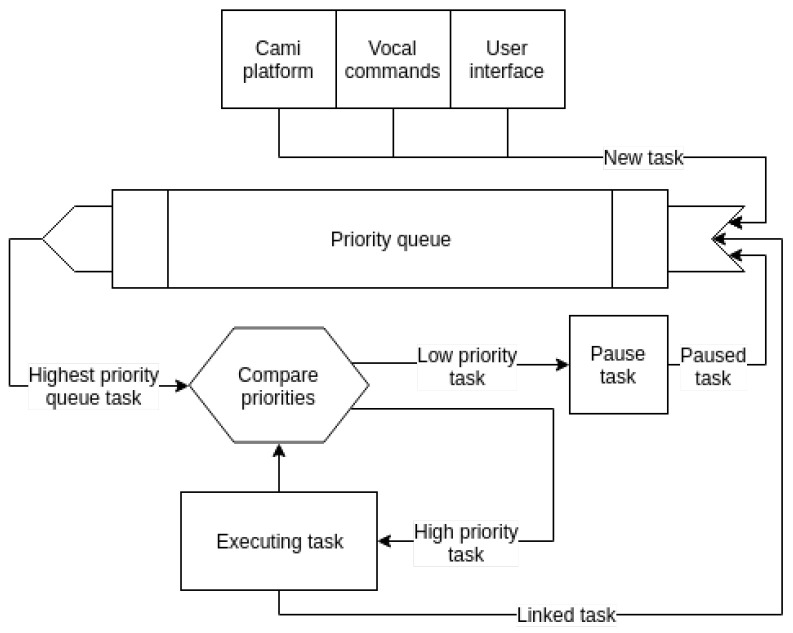
Conceptual overview of the information flow within the behavior execution module. New behaviors can be created based on events from three external or user interaction based sources: the smart environment (CAMI Platform), user voice commands (e.g., “What is my health status?”), or the user interface (on the robot tablet or an external web browser). Tasks that form the execution graph of a behavior are inserted in an execution queue based on their assigned priority. The priority enables a preemption mechanism (i.e., more urgent behaviors can pause and override currently executing ones).

Formally, the behavior composition module represents a *behavior* as a *task execution graph G*. Each node $T_{i}^{G}\left(type,priority\right)$ in the graph constitutes an *instance* of a *task*, having a *type* and a *priority* value. The *type* of the task is given by the functionality modules presented in previous sections (e.g., *Say*, *MoveTo*, *Search*—look for a given target around the robot, *Listen*—restrict speech recognition to a specific answer from the user, and *RecognizeActivity*). The *priority* value is the same for all tasks $T_{i}^{G}$ of an execution graph *G* and will influence the order in which tasks from *different* execution graphs will be scheduled for running by the task execution engine from Figure 7.

Tasks are atomic in execution, meaning that they cannot be paused. A task $T_{i}^{G}$ can end in *success* or *failure* and for each case it may be connected with another task in the execution graph *G* (e.g., $T_{i}^{G}\overset{success}{\to }T_{j}^{G}$, $T_{i}^{G}\overset{failure}{\to }T_{k}^{G}$). When a task $T_{i}^{G^{a}}$ from execution graph $G^{a}$ finishes execution (either in success or fail) its connected task (e.g., $T_{j}^{G^{a}}$ for success, or $T_{k}^{G^{a}}$ for failure) is retrieved. Let $T_{l}^{G^{b}}$ be the task with the highest priority in the priority queue. As $T_{l}^{G^{b}}$ is part of another execution graph $G^{b}$, task $T_{j}^{G^{a}}$ (or $T_{k}^{G^{a}}$ in case of failure) will be reinserted in the priority queue to be executed later, after all tasks from execution graph $G^{b}$ have ended. This mechanism enables *behavior preemption* and is a means to ensure that the robot can react to more *important* events, while still being able to complete all launched behaviors.

The current implementation has three sources of input for both simple and complex behaviors: the smart environment (the CAMI platform [59]), user voice commands (e.g., “Display my health status” and “Go to Alex”), and the web-based user interface used to test the triggering of each task.) The developer is tasked with creating handlers for the events coming from these input sources. A handler constructs the execution graph *G* that constitutes the behavior activated in response to the triggering event. We refer the reader to previous work [22] for details and examples of instantiating a complex behavior (e.g., finding a user to deliver a notification coming from the smart environment platform).

#### Integration of Behavior Planning—Future Perspective

The current behavior management method operates based on predefined plans (task sequences, together with error contingency tasks) for the specific complex behaviors that we sought to evaluate (see Section 5. However, even a conceptually simple task, such as navigating to the known (or assumed) position of a user, can become complex if the robot has to navigate in between rooms and one of the doors is locked, or its path is blocked. The navigation task must then be broken down into sub-tasks that involve logical way points (e.g., navigating from current position to room door, crossing door if open, and moving to target position in next room). Contingency situations (e.g., door closed and obstacle on path) can be solved by re-planning (such as asking for human help to remove open the door or remove the object).

In future work we plan on enabling such high-level plan decomposition in a *declarative* manner using the ROSPlan [60] framework. The method will act as an extension of the current behavior execution, by using planning and re-planning calls to create tasks to be inserted in the priority queue. Each task will be also linked to an *actionlib* (http://wiki.ros.org/actionlib/) instance. The ROS *actionlib* package adopts asynchronous communication and consists of several tools to create an action server and client that are communicating based on an action specification that defines the goal, feedback, and result messages. These messages are inserted in a *knowledge base* of the ROSPlan planning node to inform on plan execution or need of re-planning.

The ROSPlan framework demands for both the domain and problem file. It is composed of separated nodes that cooperate together to achieve a goal. The problem generator node is responsible to regenerate the problem file relying on the domain file and the knowledge base. The planning node is worked as a wrapper for several AI planner to generate the plan. We intend to integrate the POPF2 [61] planner, which is a temporal and metric planner, that can generate a temporal plan considering the duration of actions, or the distance between locations and the available paths. As the implementation is currently ongoing, we leave the experimentation and evaluation of this approach as future work.

## 4. Experiments

To assess the reliability of the functionality modules presented in Section 3, we conducted tests for each module individually using datasets collected directly from the Pepper robot (e.g., vision, depth, and audio data) in our laboratory setting. It was necessary to collect these datasets because we wanted all the evaluations to be performed using data from the robot. All these experiments performed for the proposed models using data collected using the Pepper robot have as main purpose the validation of the utility of the proposed submodules on the Pepper robotic platform. Moreover, for some modules, it was mandatory to collect data to obtain a solution capable of running in a real-time scenario made using the robotic platform. For example, if we only used benchmarks for the human actions recognition module, then the results obtained were not satisfactory. This is because most datasets are collected using static cameras, those that provide skeletal data have this data extracted using Microsoft Kinect (in the pipeline proposed by us, the coordinates are extracted using OpenPose [58] and transformed into a format similar to the one used by Microsoft Kinect), the frame rate of the cameras used to collect these datasets is higher than the of the Pepper robot.

The datasets that we collected using the Pepper robot and that were used to perform the experiments presented in this section are the following.

- *Robotic perception dataset for HAR*—we used this dataset to test the new approach proposed for the human action recognition module. We proposed and described this dataset in previous work [36], and the improvement results obtained with the current method are presented in Section 4.4.
- *Robotic dataset for coordinate estimation*—we collected this dataset to be able to estimate the coordinates of the subject relative to the robot’s position. This dataset is proposed in this paper and detailed in Section 4.2.
- *Speech dataset*—we collected this dataset to evaluate the quality of the speech recognition (for three languages) when dealing with users speech that is collected using the microphone that is integrated within the Pepper robot. Moreover, we used this dataset to evaluate our implementation of the NLU and DM components. The quality of the microphone, the linguistic accent of the user, the environmental noises, and the distance between the user and the microphone affect the results of the speech recognition. In addition, we were not able to identify datasets that cover the commands that we are targeting for the English, Romanian and French languages. Therefore, we have decided to collect our dataset using the Pepper robot. This dataset is proposed in this paper and detailed in Section 4.1.

We can provide access to these datasets upon request.

In the following we detail the experiments performed for each functionality module, describe the used performance metrics, and discuss the obtained results.

### 4.1. Speech Recognition, Command Processing, and Dialogue Management

The implementation of the dialogue module has been evaluated by 20 users: 14 users evaluated the Romanian and English languages (same users for both languages), while the French language was evaluated by 6 users.

Using voice commands, the user initiated 190 interactions for the English language, 220 interactions for the Romanian language, and 23 interactions for the French language. Therefore, a total of 5878 interactions were collected (2660 in English, 3080 in Romanian, and 138 in French).

Some samples of the ASR evaluation results are illustrated in Table 2, while Table 3 exemplifies evaluation result samples of the NLU and DM components. The intent and entities columns represent what the NLU extracted from the command of the user, the output column represents the answer of the system decided in the DM. The output column illustrates between parentheses the type of system output (visual or phonetic). The evaluation of the TTS component is currently in progress, as well as a more extensive evaluation of the dialogue module using the French language.

**Figure 8:**
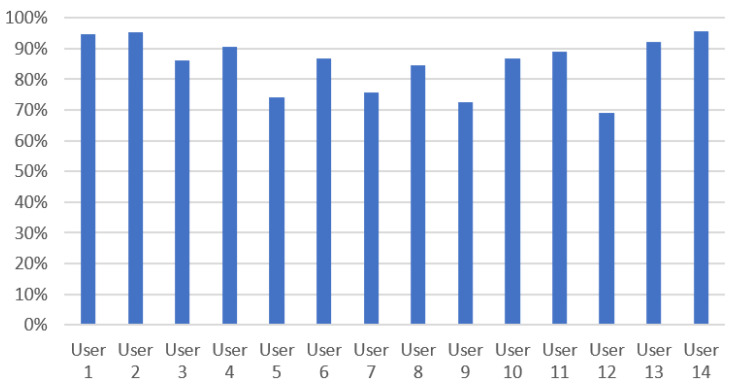
ASR Recognition percentage for each user—English language.

The overall obtained results are satisfying. In Table 2, one can observe some significant differences between the recognition accuracy of commands with similar semantics, or even similar wording. The error analysis that we performed showed that this was not caused by insufficiency of the language models for the employed ASR solution. Rather, the errors stem from a high degree of variability in the ambient noise conditions, the loudness of the speaker voice, as well as the accent in the user voice, as the ASR recognition accuracy varied significantly between the users as illustrated in Figure 8.

With respect to the intent detection objective performed by the NLU component, we show the obtained confusion matrices in Table 4, Table 5 and Table 6. The average intent detection rate is of 93.38%, 92.11%, and 80.43%, respectively, for the English, Romanian, and French languages.

### 4.2. Person Recognition and Coordinate Estimation

As the qualitative evaluation of the 3D coordinates estimation module requires the positioning error given the real position of people in the images, we created a new RGB-D dataset. The dataset consists of a collection of RGB-D images of people placed in various positions, alongside the Euler angles of the cameras at the recording moment.

We collected images from 17 people from 4 different distances, in 5 distinct postures with the cameras placed in 5 separate angles. The variety of positions provide 100 configurations for one person and for each configuration we acquired a number of 5 images. This combines into a dataset of 8500 RGB-D images (17 × 100 × 5).

As the framework requires detecting people in various scenarios, the people in the dataset were placed in 5 different postures, as follows.

(a) Standing facing the camera
(b) Standing with the back to the camera
(c) Sitting at a desk
(d) Sitting on a couch
(e) Lying on a couch

**Figure 9:**
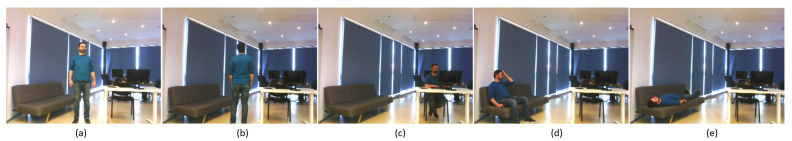
People postures used in the dataset.

Figure 9 is a visual exemplification of the 5 postures of the people.

**Figure 10:**
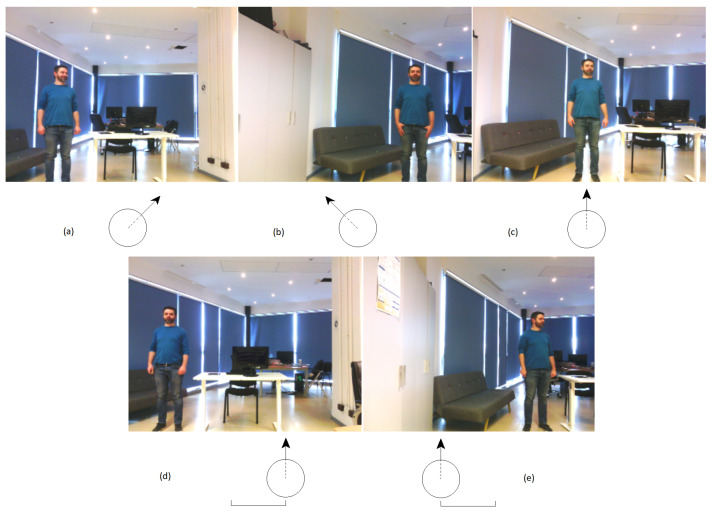
Sensors positions used in the dataset.

To change the target position in the frame, the acquisition of the images vary both in terms of camera angles and placement in the environment. The variations are comprised into 5 configurations presented in Figure 10. The rationale behind the configurations was to simulate the various situations in which the system could detect a person. The interpretation of the Figure 10 is listed below.

(a) Rotated to the right, centered
(b) Rotated to the left, centered
(c) Zero rotation, centered
(d) Zero rotation, 1 meter displacement to the right
(e) Zero rotation, 1 meter displacement to the left

In terms of distance to the target, the sensors were placed at 2, 3, 4, and 5 m away from the people, taking into consideration the maximum range of the depth sensor and the objectives of the evaluation method. Positioning people at 1 meter distance to the camera would not suit the variation of the camera angles and the postures required in the evaluation.

To assess the performance of the coordinates estimation module we computed Mean Absolute Error (MAE) and Mean Squared Error (MSE). Table 7 presents the overall errors obtained by the module on the whole dataset, while Table 8, Table 9 and Table 10 show, respectively, the errors by grouping by distance to the target, by posture of the person, and by camera position. The reported error values are expected given the accuracy of the depth sensor on the robot where the system was tested. The precision of the depth sensor is not exact and it decreases with the distance. As the values on all the three axes of the coordinates are estimated based on the distance computed by the depth sensor, the errors of the estimation are directly proportional with the accuracy of the depth sensor. Though the error is around 0.4 m, it is good enough for the purpose of the project. The output of the 3D coordinates estimation module is used as input by the navigation component in the *Move to target* action. When moving to a person, the robot does not need to match the exact location of that person, but it needs to come in his/hers proximity. Given the requirements of the project, the reported errors are satisfactory.

In terms of person detection, the error corresponds to the error obtained by the YOLO system. As the dataset does not include the ground truth bounding boxes of the people in the images, we can evaluate the precision of the detection component using a counting-based evaluation, tallying the number of detections in the images. The results of the performed evaluation are shown in Table 11, Table 12 and Table 13. By analyzing the results, the inference is that the component lacks precision when people are lying down. In all the other postures, the system performs well. The lighting conditions influence the results of the module, but do not change the results drastically. Examples for the performance of the YOLO system in different lighting conditions on images acquired by the robotic platform we use in this project can be found in [54].

### 4.3. Robot Navigation Module

**Figure 11:**
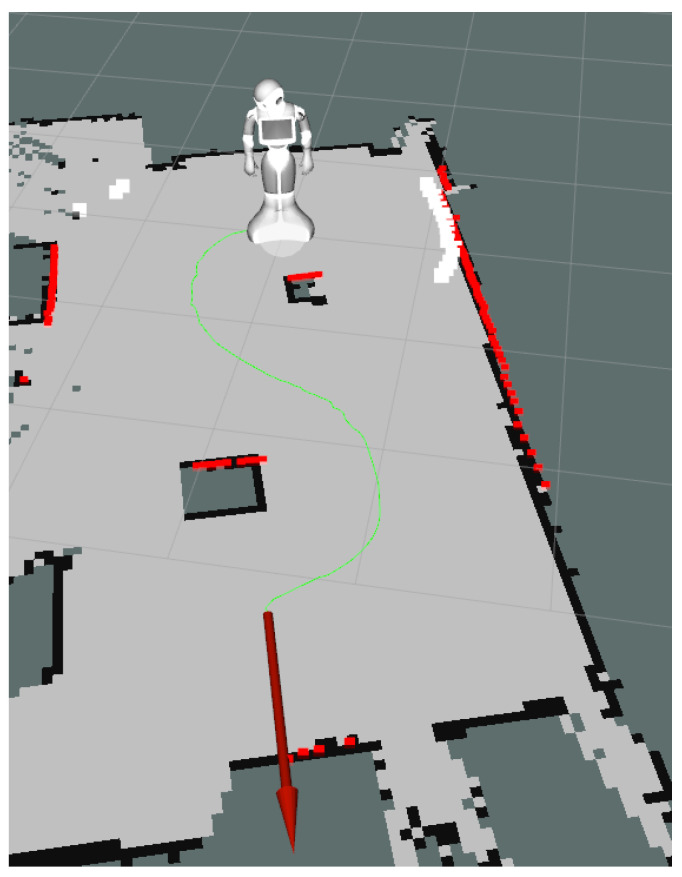
Navigation Area Path with two obstacles. The red dots are the laser scans produced by the Lidar, the white dots are the laser scans produced by the robot, the green path is the A* computed global path, and the red arrow is the goal position.

In order to have a metric-based evaluation of the navigation module, the robot was made to explore an area of 5 m × 2.5 m which was then used for three types of navigation experiments, basic forward movements, simple one obstacle avoidance, and a slalom movement between two consecutive obstacles. The first experiment was performed to show that having an external navigation module ensures that the drift can be corrected using the odometry computed by the SLAM module. The second and third experiment shows the navigation capabilities. The defined area with the robot path for the third scenario can be seen in Figure 11.

**Figure 12:**
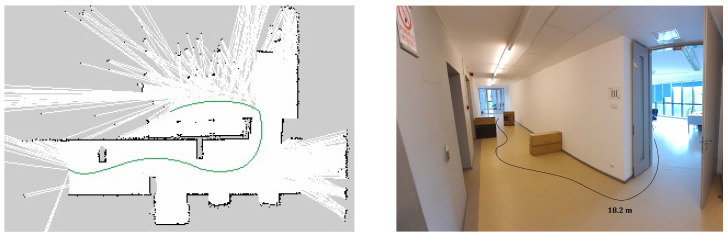
Navigation path on a larger scale experiment with obstacles placed on the hallway.

Furthermore, larger-scale experiments were performed in order to force the planner to compute new trajectories during the movement. The robot has to navigate on a 12 m long hallway in a straight line, enter through a door, turn left, and navigate forward another 4 m while maintaining its positioning. Then, obstacles were placed on the hallway in order to force the robot slalom between then in order to reach its objective. The total distance navigated by the robot is approximately 17 m when no obstacles were placed on the path and 18.2 m with obstacles. The navigation path can be seen in Figure 12.

Due to the wheel drift, the robots’ own odometry comes with large errors in both translation and rotation. The measurements performed show that the robotic platform has an error of about 10° on every 360° rotation and a drift of about 1° on any one meter forward movement. The errors can be influenced by many factors and need to be addressed in order to ensure a robust system.

The movement was controlled by the move_base ROS module using the DWA local planner [62] and A* for global planning.

**Figure 13:**
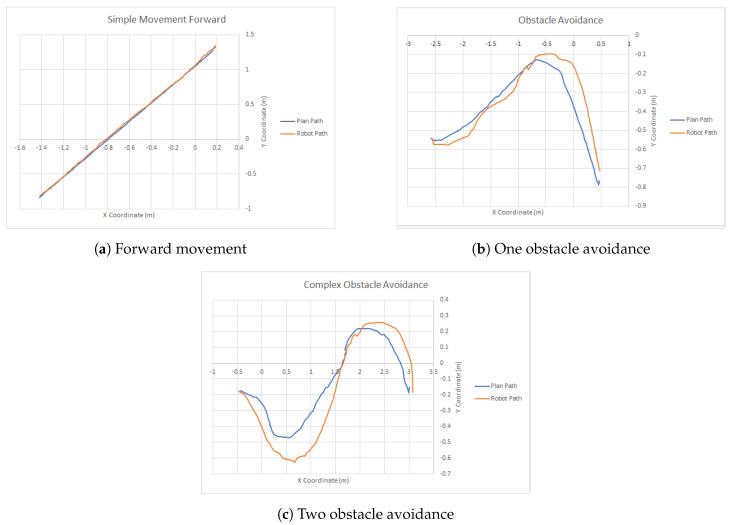
Navigation coordinate plots.

Each experiment was performed five times and the average dynamic time warping (DTW) similarity score was computed between the planned path and the actual robot movement recorded using the position computed by the SLAM algorithm. The results can be seen in Figure 13.

Figure 13a shows that the robot successfully keeps a straight line in its movement towards the goal. Due to the way the DWA local planner is tuned, the robot prefers taking larger loops when avoiding obstacles. Although this produces a longer path, it ensures smooth movements and avoids collisions between the hands of the robot and the obstacles during rotations. The results can be seen in Table 14.

**Figure 14:**
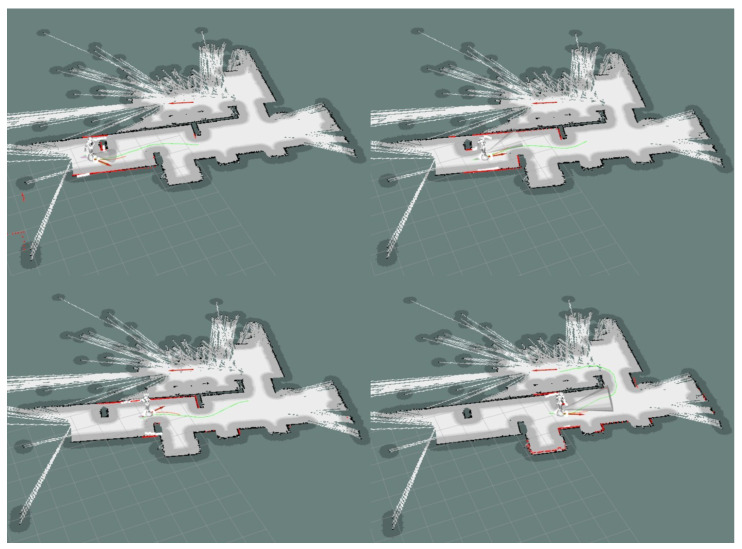
Global planner results for the larger scale experiment with obstacles on the hallway.

The experiments performed on larger-scale scenarios were repeated five times each and the average time and distance were recorded. The global planning distance is configured to 5 m. The global planning can be seen in Figure 14. If no obstacles are placed on the hallway, the robot takes an average of **54.41 s** to reach the goal for an average distance of **17.05 m**. When obstacles are used, the total distance navigated by the robot increases to **18.24 m** and it takes an average of **58.11 s** to reach the goal.

**Figure 15:**
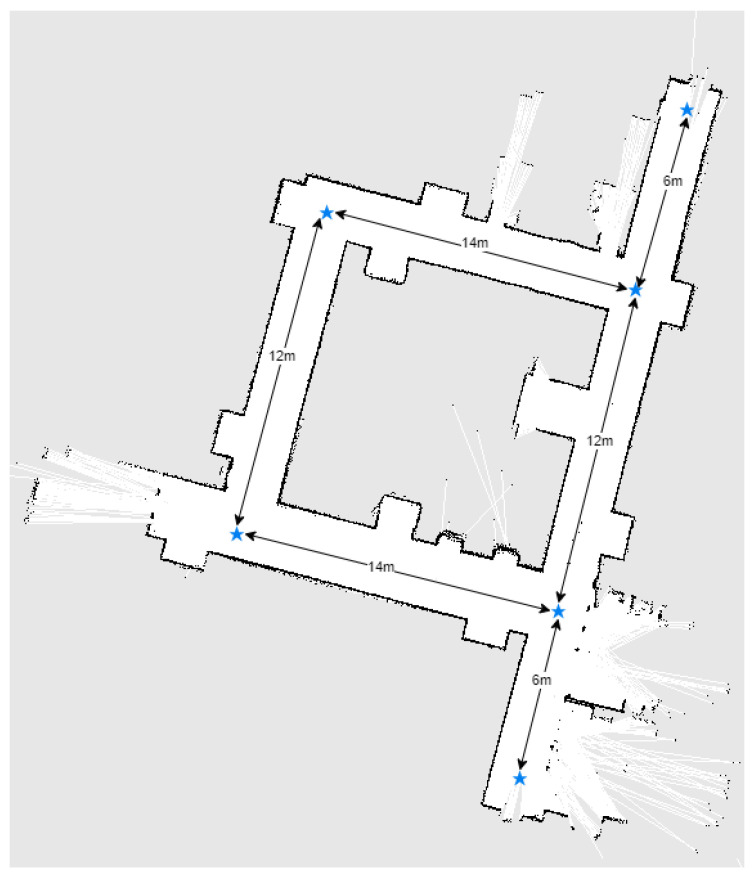
Large scale indoor mapping results. Marked with the blue stars are the goals set to the robot. The distances between goals are marked on the arrows.

Furthermore, a mapping of the entire hallway was performed while manually giving goals to the robot and can be visualized in Figure 15. The movement between goals was repeated 3 times.

### 4.4. Activity Recognition

To check if a model that is trained on a dataset recorded under normal conditions can generalize for data collected from the robot perspective, in previous work [36] we proposed a dataset collected using the Pepper robot. To create this robotic dataset, we chose 8 existing actions in the NTU RGB+D dataset [56] considered by us relevant for an assistive robot that could be used as a personal assistant. Selected actions are drink water, standing up (from sitting position), hand waving, playing with phone/tablet, typing on a keyboard, pointing at something, sneeze/cough, and touch chest (stomachache/heart pain). Considering that the results obtained for this dataset were satisfactory (presented in the Table 15), we decided to integrate an improved version in the robot’s framework to be able to detect in real-time the actions performed by the user.

As can be seen from the results obtained in Table 15, this model has a high generalization capacity, which results in poorer results for the **T1** scenario, because we no longer have a specialized solution for the examples in the NTU RGB+D dataset. However, we have a series of scenarios with better results, those that involve the use of the robotic dataset.

Given that the proposed model (Figure 6) has a large number of parameters, and we want to use it in scenarios that will run in real-time, we performed a series of performance tests. These tests were performed using a computer that has a processor with 10 cores, 20 threads, and 128GB DDR4 memory support and two RTX 2080 TI graphics cards with a memory of 11GB each. The results obtained are presented in Table 16. The measured inference time also includes the preprocessing that must be performed, using the CPU, before the data are placed in the network. To determine these results, 100 iterations were run for each scenario and an average of the results obtained for them was performed.

## 5. Evaluation Scenario

In order to test the robustness of the proposed robotic system, an evaluation scenario has been proposed and implemented. The scenario incorporates the main components of the framework illustrating a part of the basic functionalities of the integrated framework, as follows.

- voice recognition and comprehension;
- visual person finding;
- position estimation of a detected target;
- human action recognition;
- environment exploration and navigation;
- smart environment system interaction;
- actions planning and execution.

In the proposed scenario the robot interacts with a person that it needs to identify and assist in performing a basic activity, as follows.

1. The *Smart Environment and Health Management* module sends an alert to the robot about the temperature in the environment.
2. The robot initiates the visual search of the person in the environment. If the person is not identified the behavior stops.
3. The robot goes to the position of the identified person. If the person cannot be reached the behavior stops.
4. The robot announces the *hydration* notification and waits for an audio confirmation from the user.
  (a) If the confirmation is received, the *Action Recognition* module is triggered to recognize the *drink water* activity. If the action is not recognized, the robot will ask for an audio validation of the performed activity.
  (b) If the confirmation is not received, the robot will ask the user whether the *hydration* action should be postponed.
5. Pepper sends an acknowledgment to the *Smart Environment and Health Management* module.

**Figure 16:**
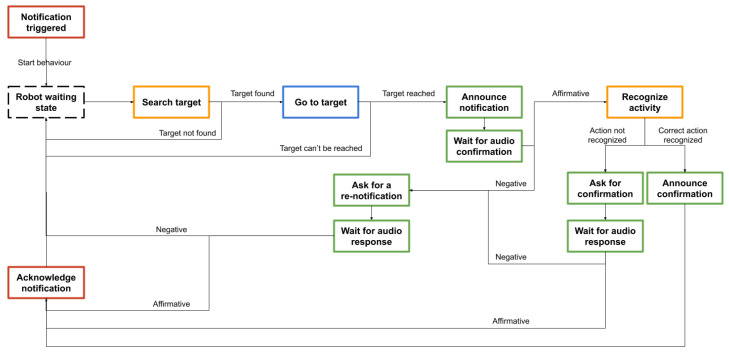
Interaction between tasks in the proposed scenario. Color coding relates to functionality modules involved in the realization of the behavior: orange—Vision module, blue—Navigation module, green—Dialogue module, and red—Behavior Composition module.

Figure 16 illustrates a visual representation of the interaction between tasks in the proposed scenario. The color scheme used in the figure indicates the components where the tasks operate: orange—*Vision* module, blue—*Navigation* module, green—*dialogue* module, and red—*Planning* module. The interaction with *Smart Environment and Health Management* component is mediated by the *Planning* component.

Due to the current situation caused by the SARS-CoV-2 virus, no experiments could be performed in real environments. Videos (http://aimas.cs.pub.ro/robin/en/rezultate/#demo) demonstrate a deployment of the scenario in the laboratory.

The scenario was tested in lab environment conditions with several people. Videos contain a part of the versions of the proposed scenario experimented with one user. The video exemplifies the general functioning of the scenario alongside the functionality of each task.

A correct execution of the scenario is observed in the video and this complex behavior was relatively easy to set up using the capabilities of the AMIRO framework. The exchange of information between the modules is working correctly, with the robot being able to detect, recognize, and localize the person in the environment alongside successful vocal interaction. The response time of the modules is adequate for a meaningful human–robot interaction and the overall user experience is satisfactory. During tests, however, we also observed a number of corner cases which need to be addressed before such a scenario can be deployed on a larger scale, in very diverse environments (e.g., user homes or various care facilities). For example, user identification by face recognition is impaired when the robot camera is directly facing a light source, potentially causing the behavior to fail, even the user them self can still be detected. This can be mitigated by enhancing the *Look for* task to include side movements of the robot base, apart from its head rotation, to avoid direct light shining into the camera.

Another observation was made concerning the activity recognition performance. During the evaluation of this scenario, we discovered a series of problems presented by the robotic system. If the robot is too close to the subject, then the skeleton predicted by OpenPose [58] is incomplete. Moreover, if there are occlusions with other objects, then the predicted skeleton is incomplete or the coordinates of some joints are incorrectly predicted. Given that the module for recognizing human activities was trained using samples in which the coordinates for all joints appeared, in such situations with a partial skeleton poor results are obtained. Also, when the robot approaches the user, the appearance in the camera may vary, leading to more difficult activity recognition. This can be mitigated in two ways: by training against a more diverse dataset, becoming more robust against joint occlusions or observation distance, as well as by enhancing the *activity recognition task* to include a forwards–backwards movement of the mobile base, so as to obtain a similar user bounding box proportion within the frame, like the ones in the dataset. These enhancements are part of our near-term future work.

## 6. Conclusions and Future Work

Social robotics is a domain with a growing interest, be it for help in the retail, entertainment, home use, or assisted living industries. Specifically, for the Active and Assisted Living (AAL) domain the economic and care-related benefits are evident [2].

However, as outlined in the motivation of the paper, one central concern of current social robotics deployments is the perception of *usefulness* and *robustness* of the provided robotic services. Users, and seniors in particular, need to have clear expectations as to the capabilities of the robotic services. When specific servicing scenarios (such as timely delivering of an important notification, monitoring correct execution of the steps in an activity, and following the user around in a home in telepresence scenarios) are exceeded to the benefit of general social abilities, the uncanny valley effect needs to be addressed. This entails ensuring the reliability of vision, natural language and context-aware functionalities in a great variety of possible deployment scenarios (e.g., single user at home and multiple users in a care facility).

In this paper, we have presented the AMIRO social robotics framework, whose objective is to address the first of the above discussed servicing paradigms: that of clear and robustly working care scenarios. We presented its platform-independent implementation based on ROS, as well as its deployment on a popular social robotics platform—the Pepper robot.

AMIRO contains functionality modules related to navigation, person detection and recognition, natural language interaction and dialogue management, activity recognition, and general behavior composition. We provided a performance metric-oriented evaluation of each functionality module, based on input coming directly from the Pepper robot. This allowed us to specifically gauge functionality limitations that are due to the Pepper robotic platform itself, or ones which can be mitigated through future improvements of our modules. We discussed the results of these evaluations, thus providing a reference of expectations with respect to the capabilities of the Pepper robotic platform to any researcher and developer that wants to use it for complex scenarios.

Furthermore, we demonstrated the deployment of all developed functionality modules in a scenario involving a specific use case—issuing health-related notifications to a user and actively monitoring their response to the notification. The scenario is executed correctly in our lab setup, as showcased in the filmed footage (http://aimas.cs.pub.ro/robin/en/rezultate/#demo), but the discussion at the end of Section 5 reveals how additional improvements are mandatory, before even such a specific scenario would be robustly enough handled to be deployed in more diverse environments.

In terms of individual functionality modules, immediate future work involves the following aspects. The set of functionality modules will be extended with the ability to perceive emotions from RGB data. The feature has already been implemented and tested separately, but is currently not integrated with the rest of the AMIRO framework, and has not been evaluated on video from Pepper’s cameras under different lighting conditions. The voice command module will be extended to include additional languages and a richer set of commands, as it will be tested out in deployment scenarios involving senior end-users from other countries. Furthermore, the proposed model for the classification of human actions can be trained so that it can predict actions even in situations where, for some frames, the detected skeleton is not complete. Extending the dataset collected with the Pepper robot for a larger number of actions is another priority we have. In addition, the 3D coordinates estimation module can be furthered improved by replacing the information coming from a depth sensor with a more reliable data source. As the values of all components of the 3D coordinate are computed based on the recorded depth, eliminating the errors in the depth data is expected to result in more accurate estimations. Last, the behavior management module will be extended in the direction explained in Section 3.7, leading to the ability to interweave predefined action sequences with planning results, based on the ROSPlan framework, thereby enabling a flexible and more extendable behavior composition functionality.

## Figures and Tables

**Table 1:** Summary of analyzed frameworks and systems in terms of their supporting platform, the core functionalities they enable, the existence of performance tests for individual functionality modules, as well as the existence of a validation through a scenario employing the whole system. The abbreviations in the Enabled functionality column refer to PD = Person Detection/Recognition, OD = Object Detection, Nav = Navigation, DM = Dialogue Management, HAR = Human Activitiy Recognition, SE = Smart Environment Integration, BM = Behavior Management.

Framework	Analysis Criteria			
	Supporting platform	Enabled functionality	Individual Functionality Testing	System Testing
NaoQI	Closed source	PD, OD, Nav, DM, BM	-	-
RADIO	Open, based on ROS	PD, OD, Nav, HAR, SE	Nav, HAR, SE	-
RAS	Open, based on ROS	PD, OD, HAR	OD, HAR	Lab deployment, activity assistance scenario
EnrichMe	Open, based on ROS	PD, OD, Nav, BM, DM	PD, OD	User home deployment, rich AAL scenario
SocialRobot	Open, based on ROS	PD, Nav, BM, DM	-	Senior center deployment, navigation and simple interaction scenarios
AMIRO	Open, based on ROS	PD, OD, Nav, HAR, DM, SE, BM	PD, Nav, HAR, DM	Lab deployment of a smart notification scenario

**Table 2:** Samples from the ASR evaluation results.

Language	User’s Command	Recognition Percentage
EN	Display my blood pressure.	92.86%
EN	Show my blood pressure.	85.71%
EN	What is my blood pressure?	92.86%
EN	How much have I walked today	85.71%
EN	How much did I walked today	78.57%
EN	Display my calendar.	100.00%
EN	What plans do I have for today?	92.86%
EN	What plans do I have scheduled for tomorrow?	78.57%
EN	How will be the weather tomorrow?	92.86%
RO	Cum stau cu sănătatea?	92.86%
RO	Afișează cât îmi este tensiunea.	85.71%
RO	Arată-mi cât îmi este tensiunea.	78.57%
RO	Ce planuri am mâine?	92.86%
RO	Cum va fi vremea mâine?	100.00%
FR	Quel est mon état de santé?	83.33%
FR	Qui es-tu?	100.00%
FR	Afficher mon calendrier.	100.00%

**Table 3:** Samples of the evaluation results for the dialogue module.

Language	User’s Command	Intent	Entities	Output
EN	Display my blood pressure.	get_health	health_entity: blood pressure output_entity: display	Your blood pressure is 126/79 mmHg. *(Visual)*
EN	What is my blood pressure?	get_health	health_entity: blood pressure output_entity: none *(default)*	Your blood pressure is 126/79 mmHg. *(Visual & Phonetic)*
EN	How will be the weather tomorrow?	get_weather	location_entity: none *(GPS_current_location)* output_entity: none *(default)*	The weather will be sunny in Bucharest. *(Visual & Phonetic)*
EN	Say how much have I walked yesterday	get_health	health_entity: walked *(GPS_current_location)* output_entity: say	Yesterday, you have walked 5167 steps. *(Phonetic)*
RO	Afișează cât îmi este tensiunea.	get_health	health_entity: tensiunea output_entity: afișează	Tensiunea dumneavoastră este 126/79 mmHg. *(Visual)*
RO	Cum stau cu sănătatea?	get_health	health_entity: sănătatea output_entity: none *(default)*	Sunteți bine. *(Visual & Phonetic)*
RO	Ce planuri am mâine?	get_calendar	dateandtime_entity: mâine output_entity: none *(default)*	Mâine aveți planificat... *(Visual & Phonetic)*
FR	Qui es-tu?	get_info	info_entity: tu output_entity: none *(default)*	Je suis Pepper, votre assistant personnel. *(Visual & Phonetic)*
FR	Quel est mon état de santé?	get_health	health_entity: état de santé output_entity: none *(default)*	Tu vas bien. *(Visual & Phonetic)*

**Table 4:** Confusion matrix for the intent detection for the English language.

EN	get_health	get_info	get_weather	get_calendar	no_detected
get_health	1419	32	0	0	5
get_info	24	386	2	1	8
get_weather	0	2	354	41	11
get_calendar	1	2	37	325	10
no_detected	0	0	0	0	0

**Table 5:** Confusion matrix for the intent detection for the Romanian language.

RO	get_health	get_info	get_weather	get_calendar	no_detected
get_health	1658	37	0	0	10
get_info	41	418	1	3	15
get_weather	1	1	394	51	11
get_calendar	3	1	48	367	20
no_detected	0	0	0	0	0

**Table 6:** Confusion matrix for the intent detection for the French language.

FR	get_health	get_info	get_weather	get_calendar	no_detected
get_health	48	4	0	0	5
get_info	2	29	0	1	2
get_weather	0	0	19	5	2
get_calendar	0	1	4	15	1
no_detected	0	0	0	0	0

**Table 7:** Overall errors of the coordinates estimation module on the dataset.

Error	Coordinates		
	x	y	z
MAE (m)	0.47	0.36	0.28
MSE (m)	0.38	0.40	0.15

**Table 8:** Errors of the coordinates estimation module by distance to the target.

Distance to Target (m)	MAE (m)			MSE (m)		
	x	y	z	x	y	z
2	0.44	0.47	0.31	0.28	0.67	0.16
3	0.22	0.43	0.27	0.09	0.58	0.14
4	0.51	0.29	0.25	0.40	0.21	0.13
5	0.72	0.26	0.30	0.74	0.13	0.16

**Table 9:** Errors of the coordinates estimation module by person postures.

Person Posture	MAE (m)			MSE (m)		
	x	y	z	x	y	z
Standing, facing the camera	0.38	0.10	0.16	0.21	0.02	0.03
Standing, back to the camera	0.38	0.10	0.14	0.21	0.02	0.03
Sitting at desk	0.56	0.43	0.21	0.51	0.32	0.09
Sitting on couch	0.52	0.60	0.21	0.47	0.84	0.06
Lying on couch	0.53	0.58	0.69	0.49	0.79	0.52

**Table 10:** Errors of the coordinates estimation module by sensors positions.

Sensors Position	MAE (m)			MSE (m)		
	x	y	z	x	y	z
Centered, no rotation	0.33	0.16	0.27	0.18	0.05	0.14
Centered, right rotation	0.52	0.58	0.26	0.48	0.83	0.14
Centered, left rotation	0.54	0.22	0.31	0.41	0.13	0.16
1 m to right, no rotation	0.51	0.54	0.29	0.46	0.79	0.16
1 m to left, no rotation	0.46	0.30	0.28	0.35	0.19	0.14

**Table 11:** Average counting errors of the detection component for the custom dataset.

**MAE**	0.18
**MAE excluding** ***lying down*** **posture**	0.03

**Table 12:** Average counting errors of the detection component by person postures.

Person Posture	MAE
Standing, facing the camera	0.00
Standing, back to the camera	0.00
Sitting at desk	0.10
Sitting on couch	0.03
Lying on couch	0.82

**Table 13:** Average counting errors of the detection component by distances.

Distance to Target (m)	MAE	MAE Excluding *Lying Down* Posture
2	0.09	0.02
3	0.16	0.03
4	0.23	0.04
5	0.22	0.04

**Table 14:** Navigation experiments  results.

	Plan Distance (m)	Robot Traveled Distance (m)	Time to Reach the Goal (s)	DTW Similarity Score
Forward movement	2.6904	2.7145	8.12	1.7803	
One obstacle avoidance	3.3636	3.4418	13.035	9.3428	
Two obstacles avoidance	3.3908	4.2177	16.034	18.5864	

**Table 15:** The results obtained for the proposed architecture.

Scenario	Accuracy	
	Cross-Subject	Cross-View
Trained on NTU RGB+D & tested on NTU RGB+D—**T1**	75.21%	79.30%
Trained on NTU RGB+D & tested on validation dataset—**T2**	60.37%	45.60%
Trained on validation dataset & tested on validation dataset—**T4**	88.67%	89.12%
Trained on NTU RGB+D & fine-tuned on validation dataset & tested on validation dataset—**T3**	**92.27%**	**91.63%**

**Table 16:** Inference time obtained for the proposed model obtained for a sequence with variable number of frames.

Sequence Length	Inference Time (*msec*)
5	6.376
10	12.435
15	18.796
20	24.689
25	30.665
30	36.925
60	72.968

## References

[1] Feil-Seifer D., Mataric M.J. Defining socially assistive robotics. Proceedings of the Rehabilitation Robotics, ICORR 2005, 9th International Conference.

[2] Payr S., Werner F., Werner K. (2015). Potential of Robotics for Ambient Assisted Living.

[3] Schiffhauer B., Bernotat J., Eyssel F., Bröhl R., Adriaans J. Let the User Decide! User Preferences Regarding Functions, Apps, and Interfaces of a Smart Home and a Service Robot. Proceedings of the International Conference on Social Robotics.

[4] Awada I.A., Cramariuc O., Mocanu I., Seceleanu C., Kunnappilly A., Florea A.M. An end-user perspective on the CAMI Ambient and Assisted Living Project. Proceedings of the 12th Annual International Technology, Education and Development Conference INTED.

[5] Pripfl J., Körtner T., Batko-Klein D., Hebesberger D., Weninger M., Gisinger C., Frennert S., Eftring H., Antona M., Adami I. Results of a real world trial with a mobile social service robot for older adults. Proceedings of the 2016 11th ACM/IEEE International Conference on Human-Robot Interaction (HRI).

[6] Wilson G., Pereyda C., Raghunath N., de la Cruz G., Goel S., Nesaei S., Minor B., Schmitter-Edgecombe M., Taylor M.E., Cook D.J. (2019). Robot-enabled support of daily activities in smart home environments. Cogn. Syst. Res..

[7] Portugal D., Alvito P., Christodoulou E., Samaras G., Dias J. (2019). A study on the deployment of a service robot in an elderly care center. Int. J. Soc. Robot..

[8] Coşar S., Fernandez-Carmona M., Agrigoroaie R., Pages J., Ferland F., Zhao F., Yue S., Bellotto N., Tapus A. (2020). ENRICHME: Perception and Interaction of an Assistive Robot for the Elderly at Home. Int. J. Soc. Robot..

[9] Pepper Robot Sales. https://bots.co.uk/pepper-robot-price/.

[10] Nap H., Suijkerbuijk S., Lukkien D., Casaccia S., Bevilacqua R., Revel G., Rossi L., Scalise L. (2018). A social robot to support integrated person centered care. Int. J. Integr. Care.

[11] Antonopoulos C., Keramidas G., Voros N.S., Hübner M., Goehringer D., Dagioglou M., Giannakopoulos T., Konstantopoulos S., Karkaletsis V. Robots in assisted living environments as an unobtrusive, efficient, reliable and modular solution for independent ageing: The RADIO perspective. Proceedings of the International Symposium on Applied Reconfigurable Computing.

[12] Fischinger D., Einramhof P., Papoutsakis K., Wohlkinger W., Mayer P., Panek P., Hofmann S., Koertner T., Weiss A., Argyros A. (2016). Hobbit, a care robot supporting independent living at home: First prototype and lessons learned. Robot. Auton. Syst..

[13] Hawes N., Burbridge C., Jovan F., Kunze L., Lacerda B., Mudrova L., Young J., Wyatt J., Hebesberger D., Kortner T. (2017). The strands project: Long-term autonomy in everyday environments. IEEE Robot. Autom. Mag..

[14] Pot E., Monceaux J., Gelin R., Maisonnier B. Choregraphe: A graphical tool for humanoid robot programming. Proceedings of the RO-MAN 2009—The 18th IEEE International Symposium on Robot and Human Interactive Communication.

[15] Glas D., Satake S., Kanda T., Hagita N. An Interaction Design Framework for Social Robots. In Proceeding of the Robotics: Science and Systems VII.

[16] Bellotto N., Fernandez-Carmona M., Cosar S. Enrichme integration of ambient intelligence and robotics for aal. Proceedings of the AAAI.

[17] Negenborn R. (2003). Robot Localization and Kalman Filters. Master’s Thesis.

[18] Groot R. (2018). Autonomous Exploration and Navigation with the Pepper robot. Master’s Thesis.

[19] Gómez C., Mattamala M., Resink T., Ruiz-del Solar J. (2018). Visual slam-based localization and navigation for service robots: The pepper case. Robot World Cup.

[20] Mur-Artal R., Tardós J.D. (2017). Orb-slam2: An open-source slam system for monocular, stereo, and rgb-d cameras. IEEE Trans. Robot..

[21] Cruz-Maya A., Garcia F., Pandey A.K. (2019). Enabling Socially Competent navigation through incorporating HRI. arXiv.

[22] Gavril A.F., Ghiță A.Ş., Sorici A., Florea A.M. Towards a Modular Framework for Human-Robot Interaction and Collaboration. Proceedings of the 2019 22nd International Conference on Control Systems and Computer Science (CSCS).

[23] Wang H., Sotnikova M.V., Korovkin M.V. Object tracking and 3D coordinates estimation using nonlinear observer for a wheeled mobile robot with a single camera. Proceedings of the 2014 2nd International Conference on Emission Electronics (ICEE).

[24] Chao C.T., Chung M.H., Chiou J.S., Wang C.J. (2016). A Simple Interface for 3D Position Estimation of a Mobile Robot with Single Camera. Sensors.

[25] Akkaladevi S.C., Heindl C. Action recognition for human robot interaction in industrial applications. Proceedings of the 2015 IEEE International Conference on Computer Graphics, Vision and Information Security (CGVIS).

[26] Song Z., Yin Z., Yuan Z., Zhang C., Chi W., Ling Y., Zhang S. (2020). Attention-Oriented Action Recognition for Real-Time Human-Robot Interaction. arXiv.

[27] Lee J., Ahn B. (2020). Real-Time Human Action Recognition with a Low-Cost RGB Camera and Mobile Robot Platform. Sensors.

[28] Rezazadegan F., Shirazi S., Upcroft B., Milford M. (2017). Action Recognition: From Static Datasets to Moving Robots. arXiv.

[29] Chiang T.C., Bruno B., Menicatti R., Recchiuto C.T., Sgorbissa A. (2019). Culture as a sensor? A novel perspective on human activity recognition. Int. J. Soc. Robot..

[30] Song Y.F., Zhang Z., Shan C., Wang L. Stronger, Faster and More Explainable: A Graph Convolutional Baseline for Skeleton-based Action Recognition. Proceedings of the 28th ACM International Conference on Multimedia.

[31] Gupta P., Thatipelli A., Aggarwal A., Maheshwari S., Trivedi N., Das S., Sarvadevabhatla R.K. (2020). Quo Vadis, Skeleton Action Recognition?. arXiv.

[32] Liu Z., Zhang H., Chen Z., Wang Z., Ouyang W. Disentangling and Unifying Graph Convolutions for Skeleton-Based Action Recognition. Proceedings of the IEEE/CVF Conference on Computer Vision and Pattern Recognition.

[33] Papadopoulos K., Ghorbel E., Aouada D., Ottersten B. (2019). Vertex feature encoding and hierarchical temporal modeling in a spatial-temporal graph convolutional network for action recognition. arXiv.

[34] Bai S., Kolter J.Z., Koltun V. (2018). An empirical evaluation of generic convolutional and recurrent networks for sequence modeling. arXiv.

[35] Kipf T.N., Welling M. (2016). Semi-supervised classification with graph convolutional networks. arXiv.

[36] Nan M., Ghiță A.Ş., Gavril A.F., Trăscău M., Sorici A., Cramariuc B., Florea A.M. Human Action Recognition for Social Robots. Proceedings of the 2019 22nd International Conference on Control Systems and Computer Science (CSCS).

[37] Trăscău M., Nan M., Florea A.M. (2019). Spatio-temporal features in action recognition using 3d skeletal joints. Sensors.

[38] Mohri M., Pereira F., Riley M. (2002). Speech Recognition with Weighted Finite-State Transducer. Comput. Speech Lang. J..

[39] Deng L., Li X. (2013). Machine Learning Paradigms for Speech Recognition: An Overview. Trans. Audio Speech Lang. Process. J..

[40] Toshniwal S., Sainath T., Weiss R., Li B., Moreno P., Weinstein E., Rao K. Multilingual Speech Recognition with a Single End-to-End Model. Proceedings of the 43rd International Conference on Acoustics, Speech and Signal Processing (ICASSP).

[41] Roark B., Saraclar M., Collins M. (2007). Discriminative n-Gram Language Modeling. Comput. Speech Lang. J..

[42] Platonov G., Kane B., Gindi A., Schubert L. (2019). A Spoken Dialogue System for Spatial Question Answering in a Physical Blocks World. arXiv.

[43] Black A.W., Schultz T., Kirchhoff K. (2006). Multilingual Speech Synthesis. Multilingual Speech Processing.

[44] Podpora M., Gardecki A., Beniak R., Klin B., Vicario J.L., Kawala-Sterniuk A. (2020). Human Interaction Smart Subsystem—Extending Speech-Based Human-Robot Interaction Systems with an Implementation of External Smart Sensors. Sensors.

[45] Perera V., Pereira T., Conell J., Velosom M. (2018). Setting Up Pepper For Autonomous Navigation and Personalized Interaction With Users. arXiv.

[46] Holthaus P., Leichsenring C., Bernotat J., Richter V., Pohling M., Carlmeyer B., Köster N., Meyer Zu Borgsen S., Zorn R., Schiffhauer B. How to Address Smart Homes with a Social Robot? A Multi-modal Corpus of User Interactions with an Intelligent Environment. Proceedings of the Tenth International Conference on Language Resources and Evaluation.

[47] Anghel I., Cioara T., Moldovan D., Antal M., Pop C., Salomie I., Pop C.B., Chifu V. (2020). Smart Environments and Social Robots for Age-Friendly Integrated Care Services. Int. J. Environ. Res. Public Health.

[48] Carolis B., Mazzotta I., Novielli N., Pizzutilo S. Social Robots and ECAs for accessing smart environments services. Proceedings of the International Conference on Advanced Visual Interfaces.

[49] Bui H.D., Pham C., Lim Y., Tan Y., Chong N.Y., Kheddar A., Yoshida E., Ge S.S., Suzuki K., Cabibihan J.J., Eyssel F., He H. (2017). Integrating a Humanoid Robot into ECHONET-Based Smart Home Environments.

[50] Nocentini O., Fiorini L., Acerbi G., Sorrentino A., Mancioppi G., Cavallo F. (2019). A survey of behavioral models for social robots. Robotics.

[51] Mori M., MacDorman K.F., Kageki N. (2012). The uncanny valley [from the field]. IEEE Robot. Autom. Mag..

[52] Redmon J., Farhadi A. (2018). YOLOv3: An Incremental Improvement. arXiv.

[53] Schroff F., Kalenichenko D., Philbin J. (2015). FaceNet: A Unified Embedding for Face Recognition and Clustering. arXiv.

[54] Ghiță A.Ş., Barbu M.Ş., Gavril A.F., Trăscău M., Sorici A., Florea A.M. User Detection, Tracking and Recognition in Robot Assistive Care Scenarios. Proceedings of the TAROS.

[55] Kohlbrecher S., Meyer J., von Stryk O., Klingauf U. A Flexible and Scalable SLAM System with Full 3D Motion Estimation. Proceedings of the IEEE International Symposium on Safety, Security and Rescue Robotics (SSRR).

[56] Shahroudy A., Liu J., Ng T.T., Wang G. Ntu rgb+ d: A large scale dataset for 3d human activity analysis. Proceedings of the IEEE Conference on Computer Vision and Pattern Recognition.

[57] Liu J., Shahroudy A., Perez M.L., Wang G., Duan L.Y., Chichung A.K. (2019). Ntu rgb+ d 120: A large-scale benchmark for 3d human activity understanding. IEEE Trans. Pattern Anal. Mach. Intell..

[58] Cao Z., Hidalgo G., Simon T., Wei S.E., Sheikh Y. (2018). OpenPose: Realtime multi-person 2D pose estimation using Part Affinity Fields. arXiv.

[59] Kunnappilly A., Sorici A., Awada I.A., Mocanu I., Seceleanu C., Florea A.M. A novel integrated architecture for ambient assisted living systems. Proceedings of the 2017 IEEE 41st Annual Computer Software and Applications Conference (COMPSAC).

[60] Cashmore M., Fox M., Long D., Magazzeni D., Ridder B., Carrera Viñas A., Palomeras Rovira N., Hurtós Vilarnau N., Carreras Pérez M. Rosplan: Planning in the robot operating system. Proceedings of the Twenty-Fifth International Conference on Automated Planning and Scheduling.

[61] Coles A., Coles A., Fox M., Long D. (2011). POPF2: A forward-chaining partial order planner. Int. Plan. Compet..

[62] Fox D., Burgard W., Thrun S. (1997). The Dynamic Window Approach to Collision Avoidance. Robot. Autom. Mag. IEEE.

